# Innovative Approaches to RC Deep Beam Strengthening: Evaluating Low-Cost Glass Fiber Wraps Against Traditional CFRP Solutions

**DOI:** 10.3390/polym17060807

**Published:** 2025-03-19

**Authors:** Panumas Saingam, Ali Ejaz, Chaitanya Krishna Gadagamma, Qudeer Hussain, Gritsada Sua-iam, Burachat Chatveera, Bodee Maneengamlert, Panuwat Joyklad

**Affiliations:** 1 Department of Civil Engineering, School of Engineering, King Mongkut’s Institute of Technology Ladkrabang, Bangkok 10520, Thailand; panumas.sa@kmitl.ac.th; 2 National Institute of Transportation, National University of Sciences and Technology (NUST), Islamabad 44000, Pakistan; enggaliejax@gmail.com; 3 Structural Engineering, School of Engineering and Technology (SET), Asian Institute of Technology (AIT), Pathum Thani 12120, Thailand; chaitugk@ait.asia; 4 Center of Excellence in Structural Dynamics and Urban Management, Department of Civil and Environmental Engineering Technology, College of Industrial Technology, King Mongkut’s University of Technology North Bangkok, Bangkok 10800, Thailand; ebbadat@hotmail.com; 5 Department of Civil Engineering, Faculty of Engineering, Rajamangala University of Technology Phra Nakhon, Bangkok 10800, Thailand; gritsada.s@rmutp.ac.th; 6 Department of Civil Engineering, Faculty of Engineering, Thammasat University, Rangsit Campus, Pathum Thani 12121, Thailand; cburacha@engr.tu.ac.th; 7 Cemkrete Co., Ltd., Bangkok 10230, Thailand; bill.bodeem@gmail.com; 8 Department of Civil and Environmental Engineering, Faculty of Engineering, Srinakharinwirot University, Nakhonnayok 26120, Thailand

**Keywords:** lightweight aggregate, deep beams, shear, low cost, strengthening, model

## Abstract

This study evaluates the performance of lightweight aggregate deep beams strengthened with low-cost glass fiber-reinforced polymer composite (Lo-G) wraps as an alternative to expensive synthetic fiber-reinforced polymers (FRPs). The investigation includes side-bonded and fully wrapped configurations of Lo-G wraps, alongside carbon FRP (CFRP) strips for comparison. The experimental results show that epoxy-based anchors provided significantly better resistance against de-bonding than mechanical anchors, improving beam performance. Strengthening with Lo-G wraps resulted in a peak capacity increase of 17.0% to 46.9% for side-bonded beams in Group 2, 10.5% to 41.4% for fully wrapped beams in the strip configuration in Group 3, and 15.4% to 42.7% for CFRP strips in Group 4. The ultimate deflection and dissipated energy were also improved, with dissipated energy increases of up to 264.6%, 322.3%, and 222.7% for side-bonded and fully wrapped Lo-G wraps and CFRP strips, respectively. The side-bonded configuration with two or three Lo-G wraps, supplemented by epoxy wraps, outperformed fully wrapped 250 mm strips in peak capacity, with peak capacity improvements of up to 46.9%. However, beams with mechanical anchors showed poor performance due to premature debonding. They rely on friction and expansion within the concrete to resist pull-out forces. If the surrounding concrete is not strong enough or if the anchor is not properly installed, it can lead to failure. Additionally, reducing strip spacing negatively impacted performance. Lo-G wraps showed an 8.5% higher peak capacity and 32.8% greater dissipated energy compared to CFRP strips. Despite these improvements, while Lo-G wraps are a cost-effective alternative, their long-term performance remains to be investigated. None of the existing models accurately predicted the shear strength contribution of Lo-G wraps, as the lower elastic modulus and tensile strength led to high deviations in prediction-to-experimental ratios, underscoring the need for new models to assess shear strength.

## 1. Introduction

Deep beams are structural components used in various applications, including building facades, transition beams, and water tank reservoirs. They are also found in geotechnical structures, underground containment systems, floor slabs for underground spaces or garages, and piling cap blocks in offshore structures [1]. Structural lightweight concrete (SLWC) offers numerous benefits. It reduces transportation costs for precast elements as lightweight materials are generally more affordable than conventional materials. In offshore structures, SLWC elements are advantageous due to their increased buoyancy and ease of towing. Additionally, SLWC provides a favorable cost-effectiveness ratio, offering both affordability and high durability. Other notable properties of SLWC include strong compressive strength, excellent fire resistance, and effective acoustic and thermal control, among others [2]. The shear strength along a plane in lightweight concrete is lower compared to that of ordinary concrete. This is due to the nature of lightweight aggregates, which allow cracks to propagate more easily through them rather than deflecting the cracks, as seen in low-strength concretes [3]. In lightweight concrete, cracking is primarily linked to the fragmentation of the aggregates, as their strength is similar to that of the matrix. As a result, the “smooth-face crack” that forms is less efficient in transmitting shear stress [4,5,6]. Studies on lightweight concrete deep beams have found that their ultimate shear strengths are lower compared to normal weight concrete [7,8,9]. Yang [8] and Yang and Ashour [9] observed that the effect of overall depth (h) on crack propagation and distribution in lightweight concrete deep beams is similar to that in conventional concrete deep beams. Yang [8] reported that the ratio of the first diagonal crack strength to the ultimate strength in lightweight concrete deep beams slightly increased as the shear-span to beam height ratio (*a*/*h*) and h increased. It was also noted that this ratio was higher in all-lightweight concrete deep beams compared to sand–lightweight concrete deep beams. Manharawy et al. [10] investigated the impact of steel fibers on the mechanical properties and crack control of lightweight foamed reinforced concrete deep beams. They explored the influence of the fiber volume, aspect ratio, and reinforcement ratio on structural behavior. Their results showed significant improvements in cracking, ultimate load, displacement ductility, and toughness due to the addition of steel fibers. Mohammad Ali and Lazim [11] produced LWC deep beams by utilizing a light expanded clay aggregate. The authors noticed a 25% reduction in the ultimate load when compared to normal weight concrete. Wu et al. investigated the shear behavior and failure modes of deep beams made from LWAC using a strut-and-tie model. They tested eight deep beams under concentrated loads, examining the effects of *a*/*h* on failure and strength. The study found that *a*/*h* significantly influenced beam strength. The authors compared their experimental results with various shear prediction models, including ACI 318-19 [12], the Tan and Cheng model [13], the softened strut-and-tie model, and the simplified softened strut-and-tie model, confirming their applicability for LWAC deep beams.

In shear-dominated regions, compression develops in one direction, while tension emerges perpendicular to it [14]. As beam depth increases, the likelihood of sudden failure due to shear behavior becomes more pronounced, especially in larger beams, which experience more extensive crack propagation compared to smaller ones [15]. Failures in deep beams typically occur due to concrete crushing in compression zones near supports or along shear cracks [16]. Khaldoun and Khaled [17] found that deep beams with a span-to-depth ratio of 2.5 exhibited some reserve strength in the post-cracking phase, leading to less brittle failure behavior. Ashour and Morley [18] emphasized the significant role of the span-to-depth ratio and reinforcement in load-carrying capacity, with horizontal shear reinforcement proving more effective than vertical reinforcement [19]. Russo et al. [20] proposed a shear strength expression for deep beams based on the strut-and-tie model, highlighting diagonal struts, longitudinal reinforcement, vertical stirrups, and horizontal web reinforcement as key factors. Additionally, Nair and Kavitha [21] compared deep beams with circular and square openings through finite element analysis, showing that beams with circular openings had superior shear resistance compared to those with square openings.

Conventional methods of strengthening the structural response include steel plate bonding, ferrocement application, or increasing cross-sectional dimensions, which have been widely used for structural rehabilitation. However, experimental research has shown that fiber-reinforced polymers (FRPs) provide significant advantages over these conventional techniques [22,23]. FRP composites are often preferred due to their lightweight properties, ease of installation, durability, high strength, resistance to corrosion, and suitability for on-site applications [24]. FRPs have become widely used in civil engineering for the strengthening and rehabilitation of reinforced concrete (RC) elements, with recent research focusing on their effectiveness in restoring structural performance [25,26,27,28,29]. Among various FRP types, carbon fiber-reinforced polymers are the most commonly used, while glass, aramid, and basalt FRPs are gaining attention for their strength, elasticity, durability, and resistance to environmental factors, enhancing energy dissipation and structural performance [30,31,32,33]. Carbon fiber-reinforced polymers (CFRPs), glass fiber-reinforced polymers (GFRPs), and aramid fiber-reinforced polymers (AFRPs) differ in mechanical properties and their effectiveness in strengthening reinforced concrete (RC) members. The CFRP has the highest tensile strength (up to 3500 MPa) and modulus of elasticity (up to 230 GPa), making it highly effective for the flexural and shear strengthening of RC beams and slabs, though it is expensive. The GFRP is more affordable and provides moderate tensile strength (1000–1500 MPa) with a lower modulus (35–50 GPa), making it suitable for corrosion-resistant applications such as bridge decks and reinforcement in marine environments. The AFRP, known for its high impact resistance and superior fatigue performance, offers intermediate tensile strength (2000 MPa) and modulus (70–125 GPa), making it effective in dynamic loading conditions like seismic strengthening [34,35,36]. The flexural strength of RC beams improves when FRP sheets or laminates are applied to the tensile zones in negative moment regions [37,38,39]. The shear strength of RC beams also improves when FRP reinforcement sheets or laminates are externally bonded to the structural member [40,41,42].

However, synthetic FRPs are expensive, energy-intensive to produce, and have high carbon emissions, with one ton of CFRPs releasing 29,500 kg of CO_2_, much more than natural fibers like hemp, which emit only 410 kg per ton [43], raising concerns about environmental and health impacts due to hazardous, non-recyclable byproducts [44,45,46]. Despite these issues, construction still accounts for 10% of global FRP use [47]. Yoddumrong et al. [48] recently proposed using low-cost glass fiber-reinforced polymer composites (Lo-Gs) for the seismic strengthening of RC columns. These composites, made from bi-directional glass fiber sheets and affordable resin, are commonly used in the boat manufacturing industry and offer a cost-effective alternative with a tensile strength of approximately 350 MPa compared to 2500 MPa for conventional GFRP and 4000 MPa for CFRP composites [49]. Lam et al. [50] showed that combining Lo-Gs with mechanical anchors enhanced the load-carrying capacity and stiffness of deep RC beams, while Lo-Gs have also been applied to improve concrete’s mechanical properties [51], enhance beam flexural performance [52], and strengthen non-ductile bridge piers [53].

The current literature review reveals that few studies have explored the response of deep beams strengthened with Lo-G wraps, making this work a novel contribution to addressing the brittle behavior of LWAC deep beams. Previous research has highlighted the issue of premature failure due to the debonding of external FRPs, prompting the use of mechanical and epoxy-based anchors in this study, with their efficiency compared. This work investigates the behavior of deep beams with different configurations of Lo-G wraps, considering the number of wraps as a parameter, and applies existing analytical models to assess their applicability in predicting the shear capacity of LWAC beams with and without Lo-G strengthening.

## 2. Experimental Program

### 2.1. Summary of Tested Deep Beams

Fourteen deep beams were tested, with parameters of interest including the strengthening configuration of Lo-G wraps, quantity of Lo-G wraps, and anchorage type. Therefore, beams were divided into four groups, as shown in Table 1. One beam in Group 1 served as the reference for the remaining beams. Six beams in Group 2 were strengthened with Lo-G wraps applied to their sides only, hereby referred to as the side-bonded configuration. Group 2 beams were further strengthened by the application of either epoxy or mechanical anchors. Beams in Group 3 were fully wrapped around their cross-section. However, the length of the full wrap along the length varied, essentially forming a strip pattern. The length of the strips was 282 mm and 50 mm. The beams were designed in such a way to induce shear failure. In the shear span, i.e., 282 mm, stirrups were not provided. At the middle and ends, close spacing, i.e., 50 mm, was provided to avoid concrete crushing. It is important to note that the 250 mm length corresponded to the complete shear span. Beams were identified by a three-part terminology. The first part “DB” identifies the deep beam, second part refers to the quantity and configuration of strengthening (i.e., G or C for Lo-G or CFRP wraps, respectively, and “SB” and “W” for side-bonded and fully wrapped configurations, respectively), and the last part denotes the anchorage type (i.e., “NA”, “MA”, and “EA” representing the cases of no anchorage, mechanical anchorage, and epoxy anchorage, respectively). For example, DB-2GW250-NA represents a deep beam strengthened with two Lo-G wraps, fully wrapped along a length of 250 mm in each shear span, without additional support from anchors. Previous works [54,55] have shown that the fully wrapped scheme produces optimal enhancement in shear compared to side-bonded or U-shaped configurations. Therefore, anchorage was not employed in fully wrapped schemes. The number of wraps is also given in Table 1.

### 2.2. Details of Deep Beams

**Figure 1:**
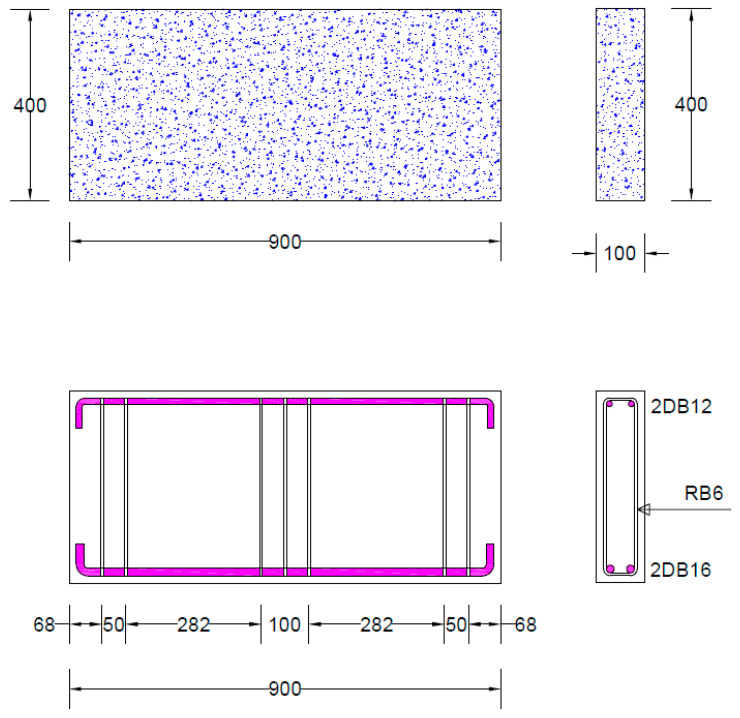
Structural details of deep beams.

All tested beams had a length of 900 mm. The depth and width of each beam were 400 mm and 100 mm, respectively. The shear span was kept at 282 mm, corresponding to a shear span-to-depth ratio of less than 1.0. In this study, 900 mm length was chosen to avoid large construction costs. Smaller concrete beams are generally easier to construct and test in laboratory settings for several reasons, particularly when considering cost efficiency. Smaller beams are lighter and easier to handle, which simplifies the construction process and reduces the need for heavy equipment. Figure 1 also illustrates the structural detailing. The tension reinforcement consisted of two deformed bars, each with a 16 mm diameter, while the top reinforcement was made up of two deformed bars, each 12 mm in diameter. Transverse reinforcement included 6 mm plain bars, which were placed only near the supports and beneath the load. Stirrups were not used in the shear spans to isolate the effect of Lo-G wraps on improving shear strength. This work did not consider the synergetic effect of Lo-Gs and stirrups. However, this may be considered in future works.

### 2.3. Material Properties

**Figure 2:**
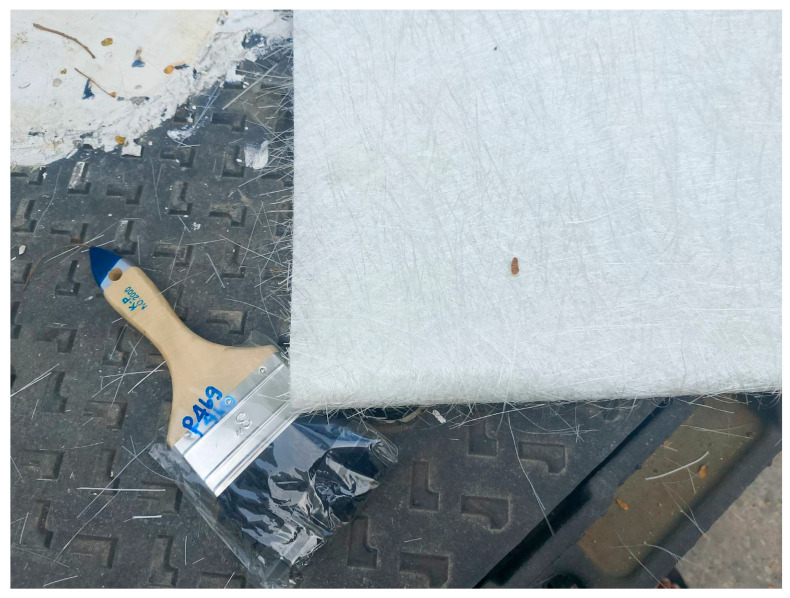
Bi-directional lo-G sheets used in this work.

The concrete was designed to achieve a target compressive strength of 25 MPa at 28 days. The yield and ultimate strengths of the bottom longitudinal reinforcement were 400 MPa and 500 MPa, respectively, while the top reinforcement showed values of 450 MPa and 550 MPa, as determined by ASTM E8 [56]. A recent study by Yoddumrong et al. [48] suggested the use of Lo-Gs to improve the seismic performance of reinforced concrete columns. Lo-Gs, commonly used in boat construction, are widely available and consist of bi-directional glass fiber sheets combined with an economical resin matrix. Known for their cost-effectiveness and adequate tensile strength, Lo-Gs present a practical material choice. The mechanical properties of Lo-G sheets were assessed according to ASTM D7565 [57], revealing an elastic modulus of 18.7 GPa, tensile strength of 377.0 MPa, and rupture strain of 2.035%. The thickness of the Lo-G sheets is 0.50 mm. Figure 2 illustrates typical Lo-G sheets, which feature randomly oriented fibers. The tensile strength of the CFRP sheets was 5241 MPa, with a thickness of 0.13 mm and fracture strain of 2.0%. Table 2 summarizes the properties of the strengthening materials. The CFRP demonstrates outstanding performance in practical applications and structural strengthening due to its high strength-to-weight ratio and durability. However, its cost remains a significant drawback, with prices ranging from approximately USD 100 to 150 per square meter per layer. Lo-G is a cost-effective alternative for structural strengthening, with prices significantly lower than CFRP. In Thailand, local suppliers offer Lo-G sheets at less than USD 3 per square meter [48], making it an affordable option for applications where moderate strength and corrosion resistance are required. It is well known that carbon fibers are primarily composed of carbon atoms arranged in a crystalline structure, which contributes to their strong covalent bonding. Glass fibers are made from silica and other materials, which do not provide the same level of atomic bonding strength as carbon.

### 2.4. Construction and Strengthening of Beams

**Figure 3:**
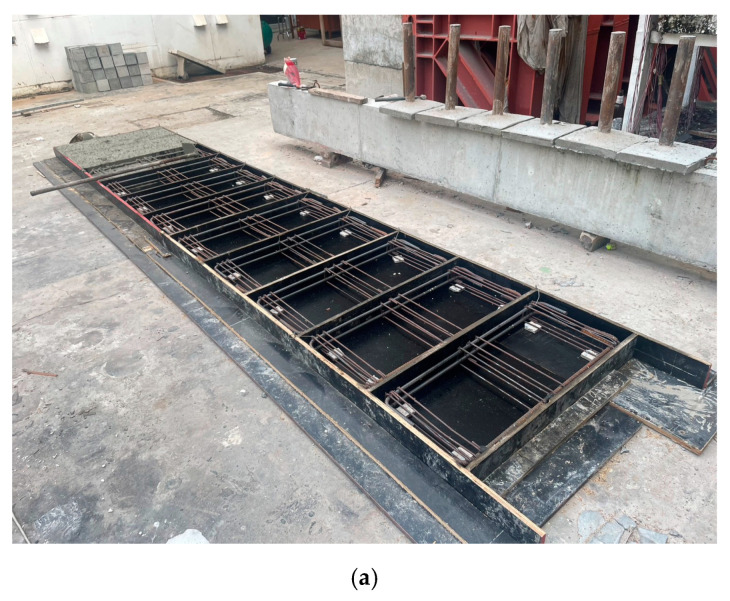

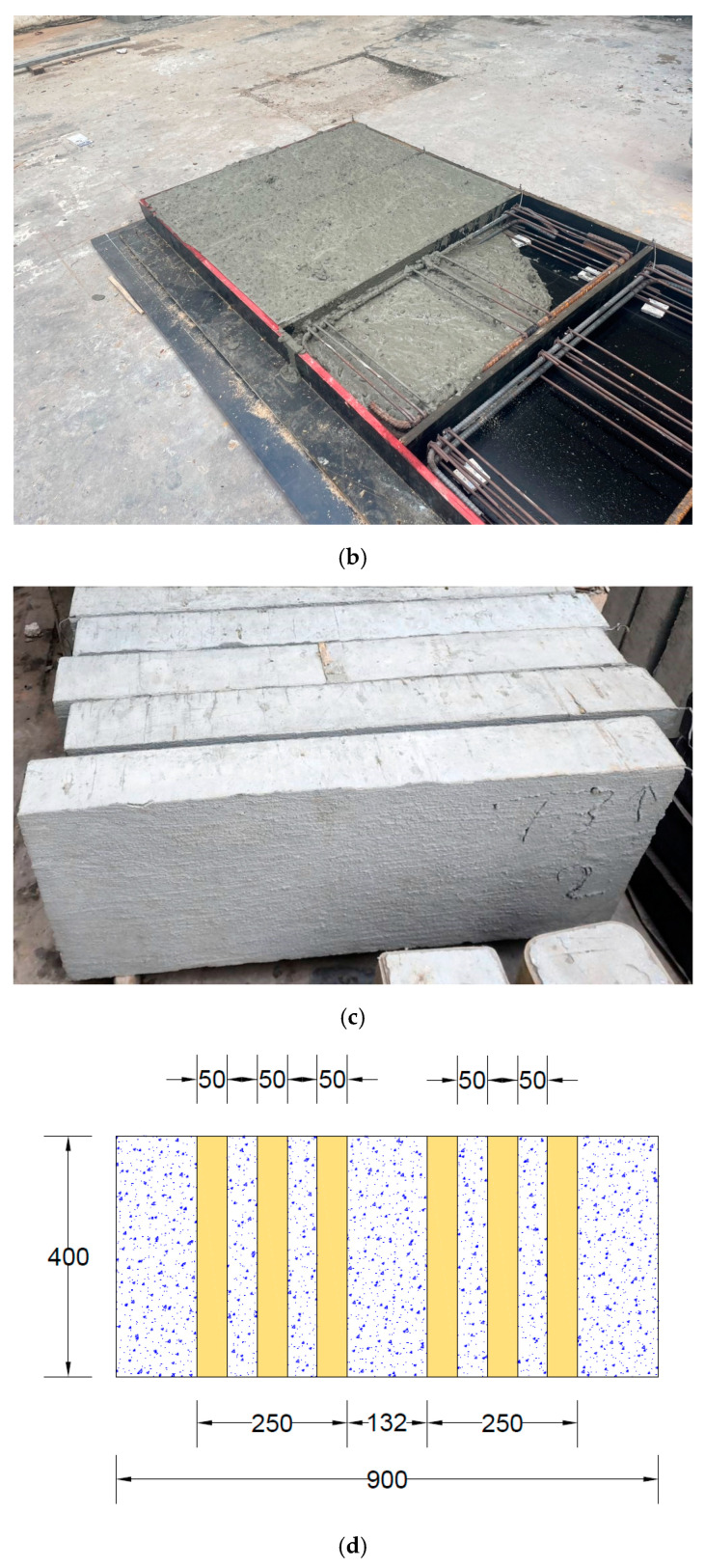

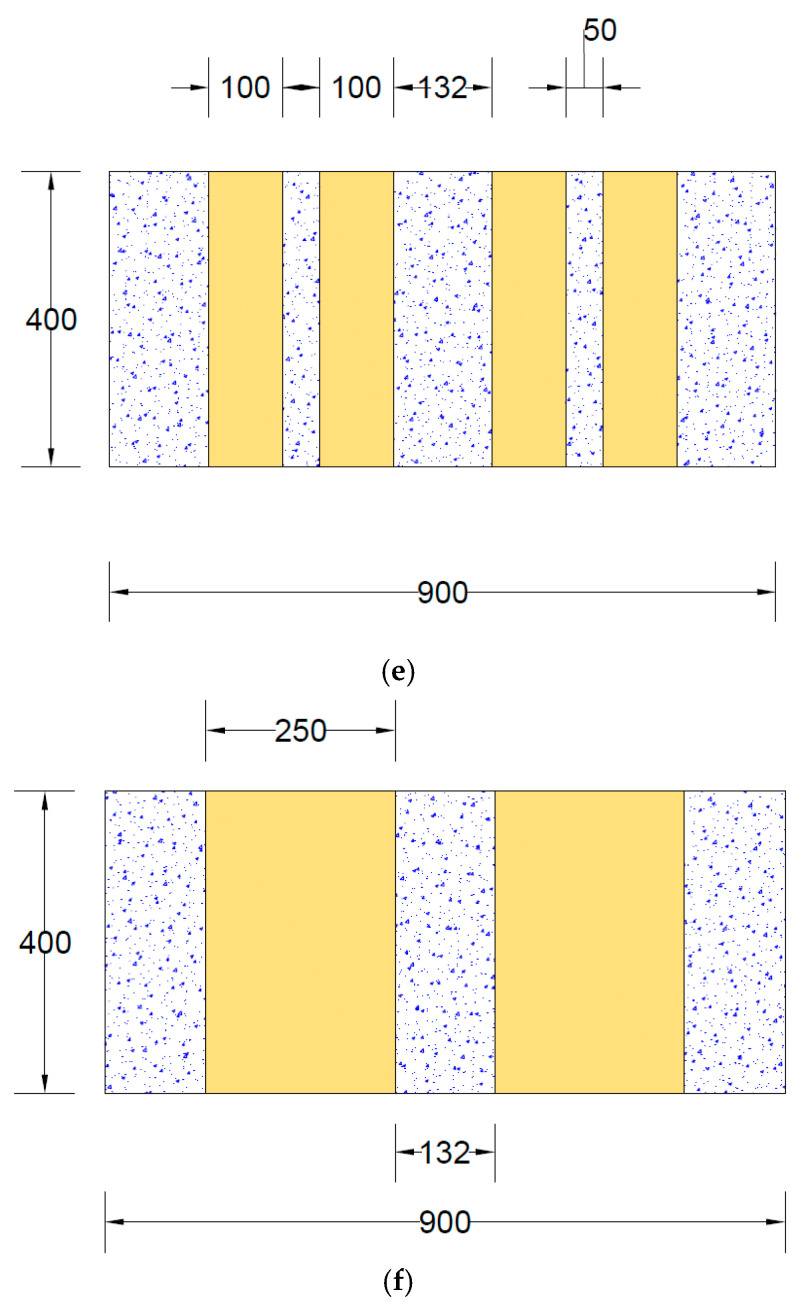
(**a**) Formwork, (**b**) concrete, (**c**) beams, (**d**) fully wrapped 50 mm strips, (**e**) fully wrapped 100 mm strips, and (**f**) fully wrapped along 250 mm length in each shear span.

**Figure 4:**
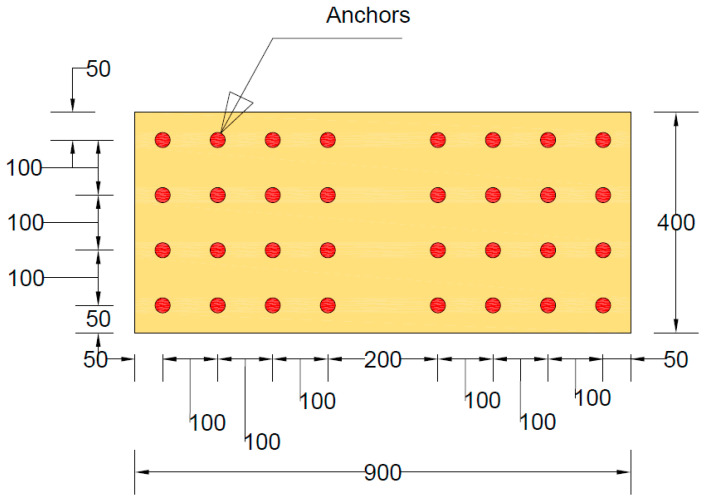
Layout of anchors for side-bonded configuration.

**Figure 5:**
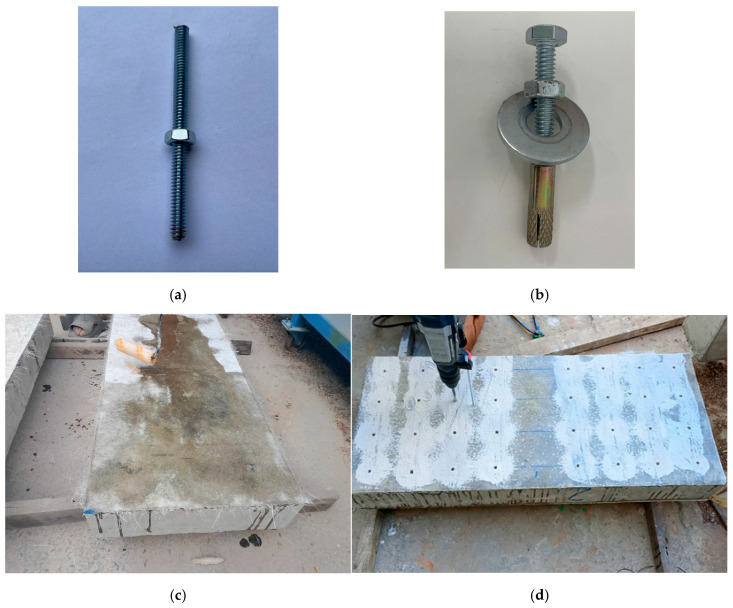

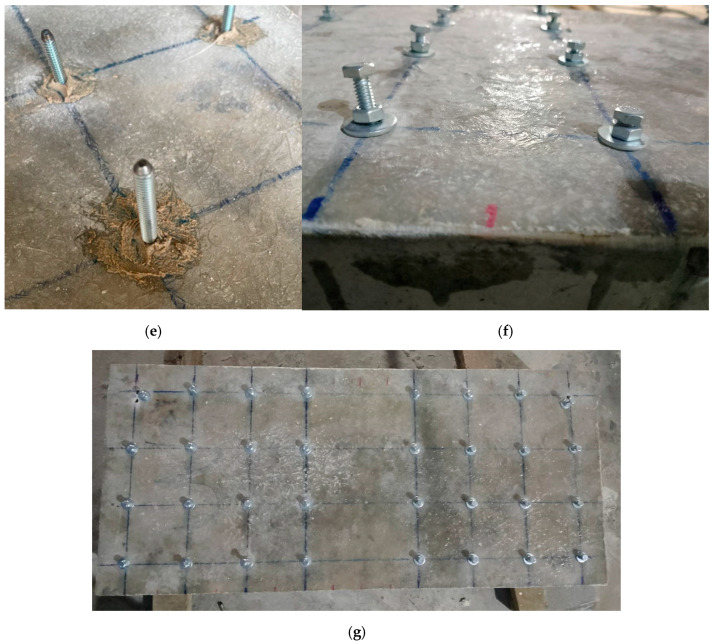
Typical (**a**) chemical and (**b**) mechanical anchor used to prevent debonding, (**c**) FRP application, (**d**) drilling, (**e**) installation of chemical anchors, (**f**) installation of mechanical anchors and (**g**) typical FRP strengthened and anchored specimen.

The steel cage was initially assembled outside of the formwork. Spacers were carefully placed inside the formwork to ensure the steel cage was positioned with a uniform clear cover of 16 mm on all sides. Square and rectangular Styrofoam inserts were incorporated into the cage to create openings. During the casting process, the beams were placed horizontally while the concrete was poured. After 28 days of curing, the beams were reinforced using the Lo-G confinement technique. This confinement was applied specifically to the shear spans of the beams, as shown in Figure 3. It is noteworthy that the side-bonded configuration was applied along the full length of beams. Figure 4 presents the layout of the anchors applied on side-bonded configuration. As described earlier, two types of anchors were used in this study. The installation of mechanical anchors followed these steps: Initially, the wet layup method was used to bond the strengthening sheets to the concrete surface. Epoxy was applied to the concrete substrate using a hand roller, and FRP sheets were then attached. After that, holes were drilled into the slabs and cleaned thoroughly. The golden section of each anchor (shown in Figure 5) was then placed into the drilled holes. Finally, a bolt and washer were inserted into the opening of the golden section and tightened. The wet layout method [58] was used to apply the Lo-G sheets. The beam surfaces were thoroughly cleaned and coated with polyester resin to promote adhesion. The first layer of Lo-G sheets was applied and saturated with resin. For beams requiring two layers of Lo-G, this process was repeated to ensure proper strengthening.

### 2.5. Test Setup

**Figure 6:**
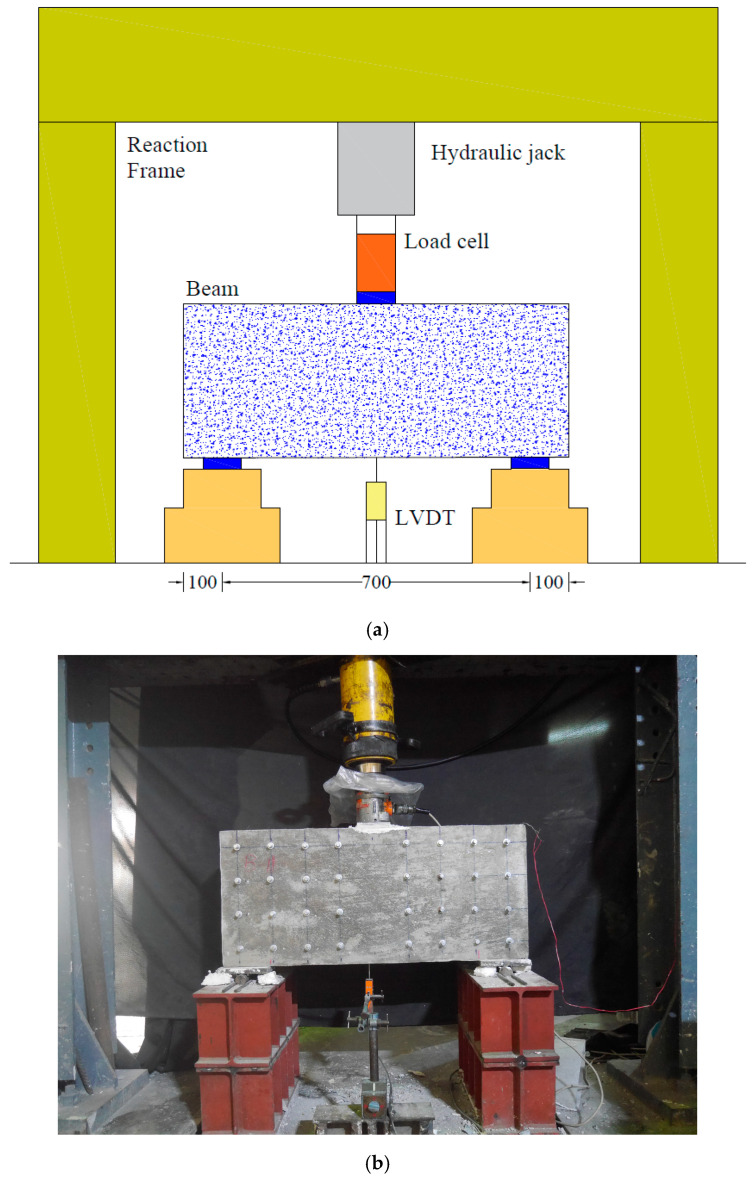
Three-point bending test setup (**a**) schematic and (**b**) system installed in lab.

Figure 6 depicts the test setup, where each beam underwent a three-point bending test until failure. The clear span of the beams was maintained at 700 mm. Deflection at the mid-span was monitored using a displacement transducer placed at the center. Steel plates, each 25 mm thick and 150 mm wide, were positioned beneath the loading point and above the supports to ensure even load distribution. The formation and propagation of cracks were closely observed and documented, and the failure mode was recorded. A hydraulic jack with a 500 kN capacity was used to apply the load incrementally. A load cell measured the applied load at each stage, while a data logger simultaneously recorded the load and corresponding beam deflection. A strain gage was also attached to the bottom longitudinal bar at midspan.

## 3. Experimental Results

### 3.1. Failure Patterns of Deep Beams

**Figure 7:**
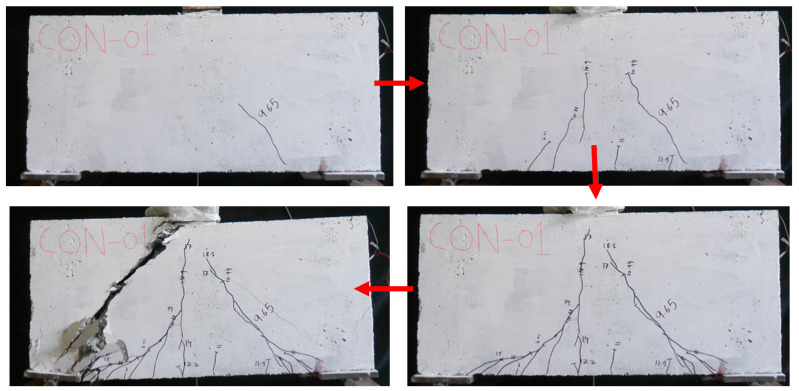
Failure of the control deep beam.

The failure mode of the beam in Group 1 is shown in Figure 7. The control beam, DB-Ctrl, exhibited a sudden and abrupt failure, which is typical of a shear-dominated mechanism. Initially, vertical cracks appeared at the bottom of the beam and gradually extended upward, changing direction to form an oblique angle. Eventually, the crack on the right-hand side widened significantly and extended toward the loading point. This led to the beam failing suddenly, with minimal vertical deflection observed prior to failure. The major diagonal crack pattern (Figure 7) indicates that the load was primarily supported by the compressive strut, consistent with findings by Yang et al. [59].

**Figure 8:**
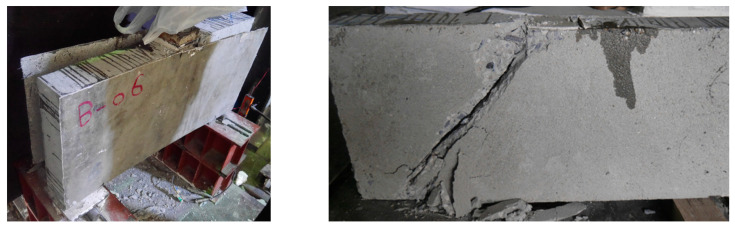
Failure of beam DB-2GSB-NA.

**Figure 9:**
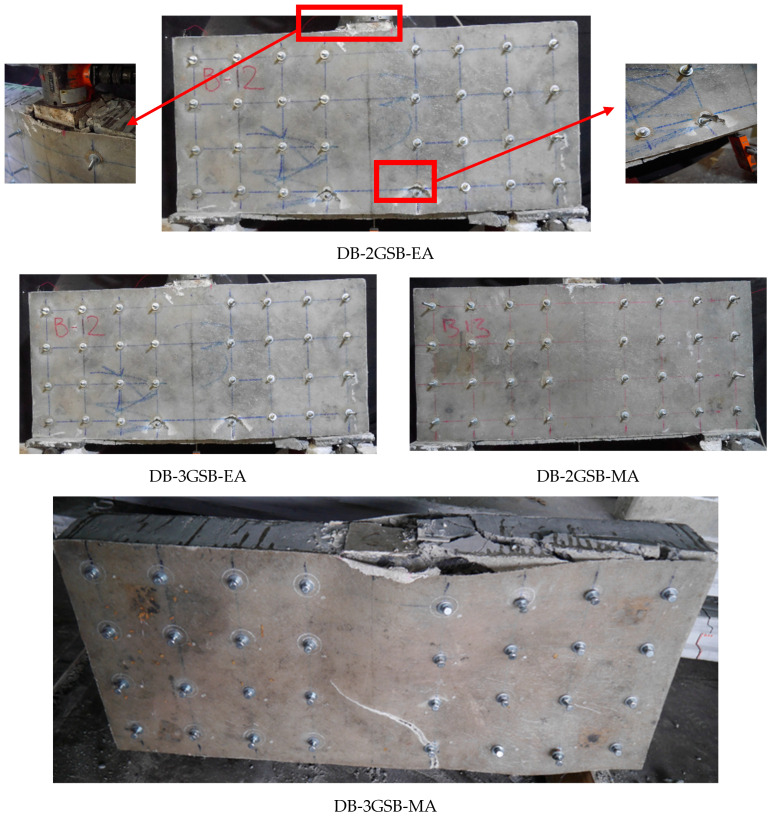
Failure of beams in Group 2.

The failure of beam DB-2GSB-NA (the Group 2 beam strengthened in a side-bonded configuration without anchors) is shown in Figure 8. This beam demonstrated the debonding of Lo-G wraps. It is further shown in Figure 8 that the failure was once again controlled by the formation of a compressive strut, analogous to the control beam DB-Ctrl. This reflects the importance of preventive measures to prevent debonding. The failure of beams in Group 2 (with anchor support) is shown in Figure 9. Typically, these beams showed concrete crushing under the load, as highlighted in Figure 9. The fracture of Lo-G wraps was not observed. Interestingly, the debonding of Lo-G wraps around midspan was observed. This may be ascribed to the reduced anchorage effect, as anchors were concentrated mainly near the supports.

**Figure 10:**
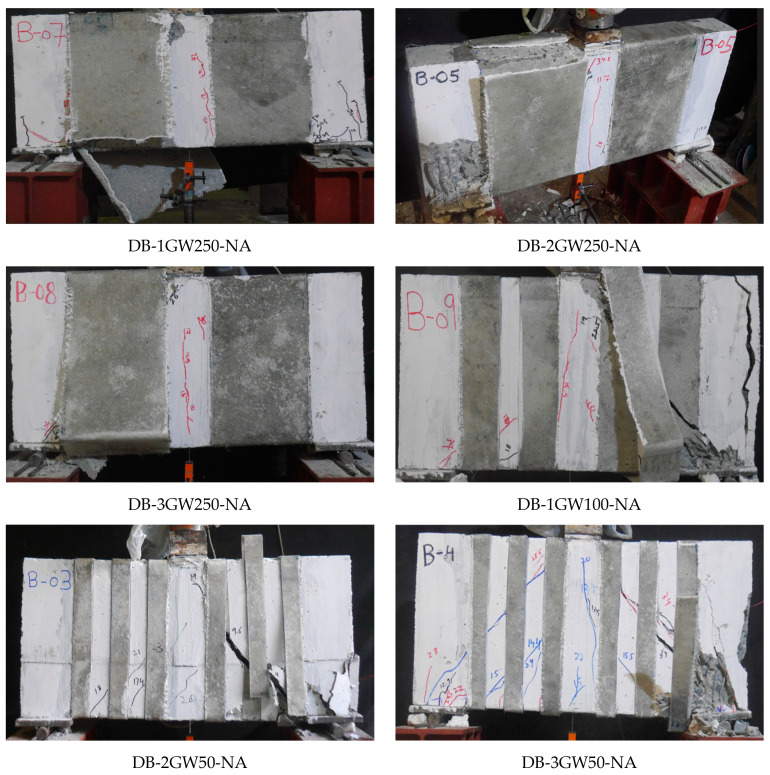
Failure of beams in Group 3.

**Figure 11:**
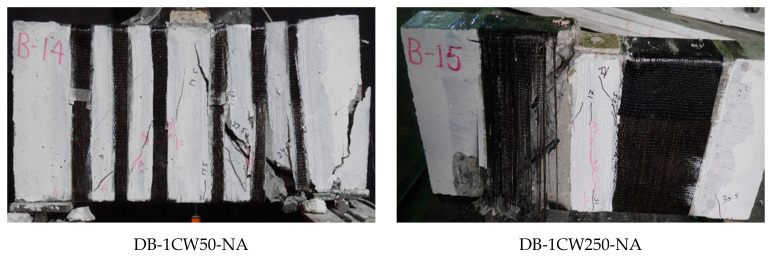
Failure of beams in Group 4.

Figure 10 shows the failure of Group 3 beams. These beams were fully wrapped around the perimeter of beams in a strip configuration, with differences in the strip width and spacing. All beams in Group 3 demonstrated the fracture of Lo-G wraps, mainly near the corners of the section, as shown in Figure 10. After removing the wraps, it was noticed that the formation of a compressive strut was inevitable, even for the case of three Lo-G wraps, i.e., DB-3GW250-NA. Figure 11 shows the failure of beams strengthened with CFRP strips, showing similar failure patterns to those strengthened with Lo-G wraps. It is noted that all beams in Groups 3 and 4 showed the fracture of the confinement and concrete crushing under the load and near supports. In the strengthened beams, the strips effectively intersected and restrained the progression of inclined shear cracks, resulting in a substantial improvement in their load-carrying capacity [60]. It is important to note that as concrete cracks begin to open, the debonding of the FRP from the concrete is likely to occur to ensure deformation compatibility between the two materials; otherwise, the FRP would need to develop unrealistically high strains at the crack location. However, this debonding is expected to remain localized [61].

### 3.2. Load vs. Deflection Response

**Figure 12:**
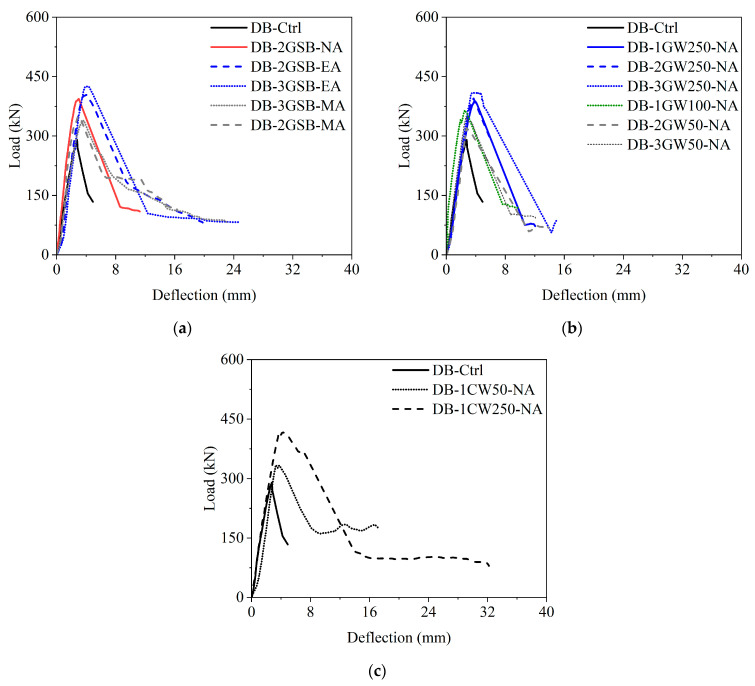
Load vs. deflection response of beams compared to the control beam: (**a**) Group 2, (**b**) Group 3, and (**c**) Group 4.

The load vs. deflection response of beams in Group 2 is compared with the control beam, as shown in Figure 12a. The strengthened beams showed an enhanced peak load capacity. Some interesting observations were made: (1) The beam strengthened without anchors showed greater strength than beams strengthened with mechanical anchors. This can be ascribed to the poor anchorage provided by mechanical anchors, which led to the premature debonding of Lo-G wraps. This further illustrates the need for an anchorage system that should at least be stronger than the bond attained by resin in the wet layup process. (2) The number of Lo-G wraps positively influenced the peak capacity, as beams strengthened with three Lo-G wraps showed greater capacity than beams strengthened with two Lo-G wraps. (3) The initial stiffness of beams was not significantly changed after strengthening. (4) The performance of beams strengthened with epoxy anchors was superior to beams without anchors and beams with mechanical anchors (the mechanical anchors rely on friction and expansion within the concrete to resist pull-out forces. If the surrounding concrete is not strong enough or if the anchor is not properly installed, it can lead to failure. Additionally, reducing strip spacing negatively impacted performance. (5) The rate of the post-peak strength degradation was similar in the strengthened and control beams. The last observation is important in the sense that despite positively influencing the peak capacity, anchors could not sustain the debonding forces and the beams ultimately failed due to a combination of compressive struts and the debonding of Lo-G wraps. The comparison of the load vs. deflection response of Group 3 beams with the control beam is shown in Figure 12b. Clearly, all the strengthened beams showed improved capacity. This was expected since the full wrapping around the perimeter of the beams resulted in failure dominated by the rupture of Lo-G wraps. Finally, Figure 12c shows the performance of beams strengthened with CFRP wraps, showing an improvement in the peak capacity, whereas the post-peak strength degradation rate was not affected. It is noted that despite the capacity improvement showing a positive correlation with the number of Lo-G wraps, future research should focus on a greater number of wraps to assess the upper limit, if any, that could be applied to the improvement in shear capacity.

### 3.3. Peak Capacity, Ultimate Deflection, and Dissipated Energy

The peak load and ultimate deflection results are summarized in Table 3. Significant improvements were observed in both parameters. The ultimate deflection was defined as the deflection corresponding to a 20% reduction from the peak load. The ultimate deflection was defined as the deflection corresponding to a 20% reduction from the peak load to provide a consistent and objective measure of post-peak behavior. This criterion is commonly used in structural performance assessments to capture the residual capacity and ductility of strengthened beams before significant degradation occurs. The 20% drop threshold ensures a standardized comparison across different strengthening configurations while avoiding excessive deformation that may not be structurally meaningful. Similar definitions have been used in previous studies [62,63] on FRP-strengthened RC beams to evaluate post-peak ductility and energy dissipation. It is seen that the peak capacity improved by the application of Lo-G wraps. For the side-bonded configuration in Group 2, the peak capacity improved by 17.0% to 46.9%, with this improvement related to both the quantity of Lo-G wraps and anchorage type. In Group 3, for fully wrapped beams in the strip configuration, the improvement ranged from 10.5% to 41.4%. Finally, CFRP strips in Group 4 demonstrated a 15.4% and 42.7% improvement in the peak capacity for 50 mm and 250 mm strips, respectively. Besides improving the peak capacity, the objective of Lo-G wraps was to delay the failure by enhancing ductility. The dissipated energy was calculated by estimating the area under the load vs. deflection curve of each beam. Importantly, the area was calculated till the ultimate deflection. The dissipated energy improvement is given in Table 3. Compared to the peak capacity and ultimate deflection, the improvement in dissipated energy was largest. Up to 264.6%, 322.3%, and 222.7% improvements in dissipated energy were reported in Groups 2, 3, and 4, respectively.

### 3.4. Effect of Strengthening Configuration

**Figure 13:**
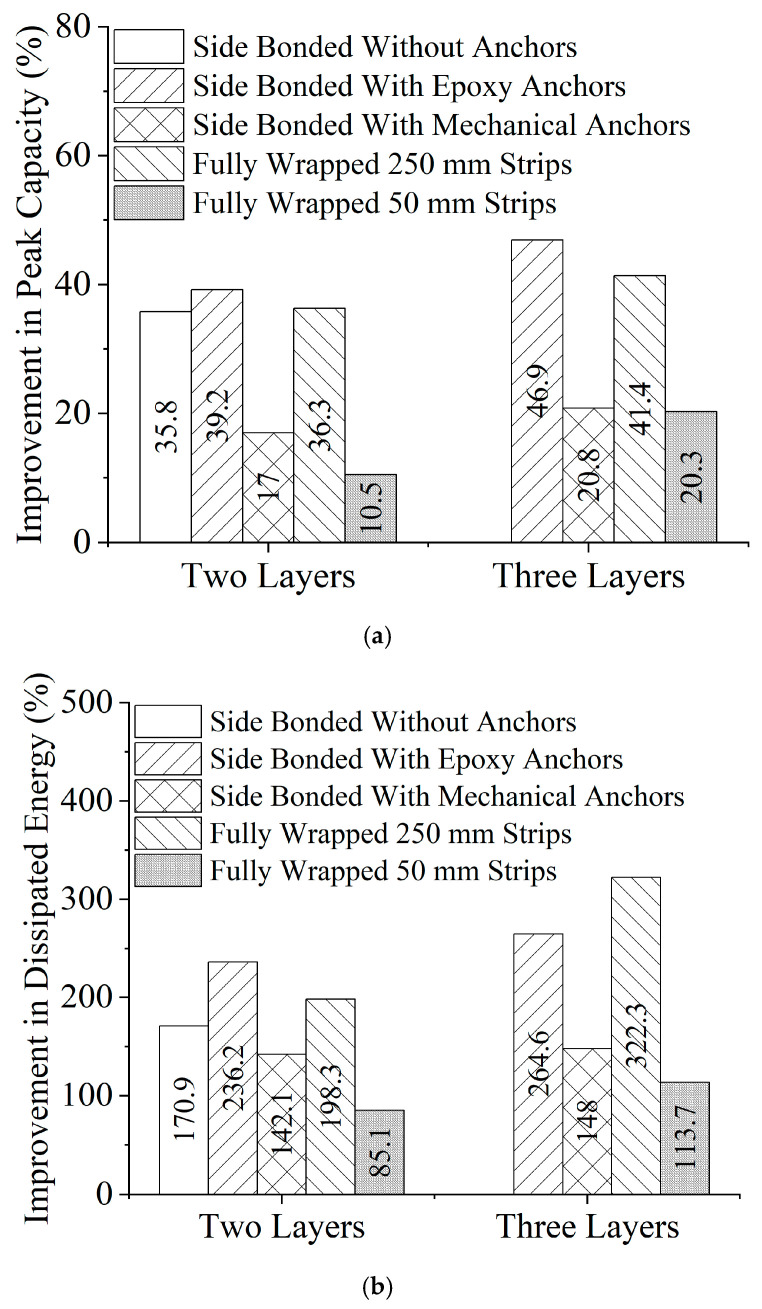
Effect of the strengthening configuration on improvement in (**a**) peak capacity and (**b**) dissipated energy.

Previous works have identified the debonding mechanism of external wraps, especially in side-bonded configurations [64,65]. The failure modes of deep beams in Group 2 confirmed that, indeed, the side-bonded configurations of Lo-G wraps are prone to debonding. The effect of debonding in the side-bonded configuration is evident by comparing the peak capacity improvement of beams DB-2GSB-NA and DB-2GSB-EA, demonstrating improvements of 35.8% and 39.2%, respectively, as shown in Figure 13a. A similar effect was reflected in dissipated energy, as shown in Figure 13b, with 170.9% and 236.2% improvements for beams DB-2GSB-NA and DB-2GSB-EA, respectively. It is important to note that the side-bonded configuration with either two or three wraps and supplemented by epoxy wraps resulted in peak capacity improvements larger than that imparted by fully wrapped 250 mm strips. Notably, the performance of Lo-G wraps with mechanical anchors was subpar, ascribed to (1) the poor anchorage performance of mechanical anchors that resulted in premature debonding and (2) the reduction in the cross-sectional area after the pull-out of mechanical anchors. The comparison further illustrates that reducing the strip spacing to 50 mm was detrimental as it significantly reduced the performance of Lo-G wraps.

### 3.5. Effect of Strengthening Type

**Figure 14:**
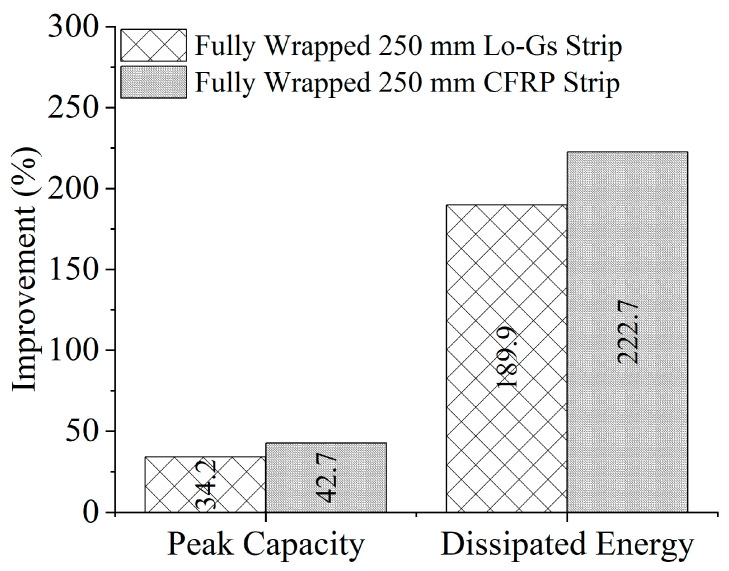
Effect of strengthening type on improvement in performance of deep beams.

A comparison of the strengthening type on peak capacity and dissipated energy improvement is shown in Figure 14. The comparison is made for 250 mm strips, with CFRP strips slightly edging past Lo-G wraps. The peak capacity and dissipated energy were 8.5% and 32.8% greater for beams strengthened with CFRP strips than Lo-G wraps. Given the cost difference between Lo-Gs and CFRPs, it is safe to comprehend that Lo-Gs are an effective alternate to CFRP strips. However, the long-term performance of Lo-G wraps is yet to be investigated.

## 4. Performance Evaluation of Existing Model Expressions

### Design Expressions for FRP Contribution to Shear Strength

This part assesses the performance of existing FRP models to predict the shear strength contribution by FRP strengthening. Since this is a novel application of Lo-Gs in strengthening the shear capacity of deep beams, the analytical assessment of existing models for Lo-G strengthening has not been explored before. The overall shear strength $V_{t}$ of reinforced concrete (RC) beams strengthened with fiber-reinforced polymers (FRPs) is typically determined by combining the contributions of the concrete $V_{c}$, the transverse reinforcement $V_{s}$, and the FRP $V_{f}$. As no shear reinforcement was included, the term $V_{s}$ was disregarded. Consequently, the experimental contribution of Lo-G wraps to the total shear strength was calculated by subtracting the shear capacity of the control beams from the total shear capacity of the strengthened beams.

(1) $$V_{t}=V_{c}+V_{f}$$

Khalifa et al. [66] suggest that the shear strength contribution of FRPs in a deep beam can be calculated using the following expression:

(2) $$V_{f}=\rho_{f}E_{f}\epsilon_{fe}b_{w}d_{f}\left(1+cot\beta \right)sin\beta$$

where $E_{f}$ denotes the elastic stiffness of FRP, $d_{f}$ is the depth of FRP, $\epsilon_{fe}$ is the effective rupture strain capacity of FRP, and $\rho_{f}$ denotes the volumetric ratio of FRP. The term $b_{w}$ denotes the width of the section, while β is an angle that comes into play in the case of inclined FRP application. The volumetric ratio of FRPs is determined by

(3) $$\rho_{f}=\frac{2n_{f}t_{f}w_{f}}{b_{w}s_{f}}$$

where the volumetric ratio involves the thickness, quantity, width, and spacing of the FRP represented by $t_{f}$, $n_{f}$, $w_{f}$, and $s_{f}$, respectively.

Triantafillou [67] demonstrated that the effective strain in externally bonded FRP sheets or strips depends on their axial rigidity ($\rho_{f}E_{f}$). Using experimental data, the effective strain was derived by back-calculating from the observed shear strength contribution ($V_{f}$), and an empirical relationship was established by correlating the effective strain with axial rigidity based on test results from 40 beams documented in earlier studies. Building on this methodology, Khalifa et al. [66] analyzed a larger dataset of 48 beams, encompassing two types of FRP materials (carbon and aramid), three wrapping configurations (side-only, U-shaped, and full wraps), and both continuous and strip forms of FRPs. Khalifa et al. [66] proposed three equations for calculating the reduction factor ($R_{f}$) and recommended using the smallest value to estimate the effective strain ($\epsilon_{fe}$). This strain was subsequently used in the shear strength calculations of FRP-strengthened beams, as outlined in Equation (2). Although these equations were developed considering both FRP rupture and de-bonding failure modes, Khalifa et al. [66] advised using Equation (4) primarily for cases involving FRP rupture.

(4) $$R_{f}=0.5622{\left(\rho_{f}E_{f}\right)}^{2}-1.2188\rho_{f}E_{f}+0.778$$

Khalifa et al. [66] highlighted the significance of limiting the shear crack width by setting an upper limit of 0.5 for the reduction factor ($R_{f}$).

In 2002, Triantafillou and Antonopoulos [67] introduced three regression-based equations based on the analysis of 75 experimental datasets. Two of these equations were designed for CFRP wraps, while the third was specifically formulated for fully wrapped aramid FRP wraps. The equation for the fully wrapped CFRP wrap is expressed as

(5) $$\epsilon_{fe}=0.17{\left(\frac{f_{c}^{\frac{2}{3}}}{\rho_{f}E_{f}}\right)}^{0.30}\epsilon_{fu}$$

The contribution of FRPs to the overall shear strength is obtained by substituting the effective strain calculated using Equation (5) into Equation (2).

In 2005, Zhang and Hsu [68] proposed two alternative equations for determining the reduction factor ($R_{f}$). One of these equations incorporated the effect of concrete strength and was derived through regression analysis of experimental data, as shown below:

(6) $$R_{f}=1.4871{\left(\frac{\rho_{f}E_{f}}{f_{c}}\right)}^{-0.7488}$$

Zhang and Hsu [68] further developed an analytical expression for the reduction factor ($R_{f}$) by conducting a detailed analysis of the bonding mechanism. This expression is given as

(7) $$R_{f}=\frac{\tau_{max}L_{e}}{2}f_{fu}t_{f}\leq 1\;$$

Here, $\tau_{max}$ represents the ultimate direct shear strength, which is calculated using Equation (8), and $L_{e}$ is assumed to be 75 mm.

(8) $$\tau_{max}=\left(7.64\times 10^{-4}f_{c}^{2}\right)-\left(7.64\times 10^{-2}f_{c}\right)+6.38\;$$

The minimum $R_{f}$ value obtained from Equations (6) and (7) is used to calculate the effective tensile strain in the FRP. Additionally, Zhang and Hsu [68] recommended a maximum $R_{f}$ value of 0.4. They also proposed an equation similar to Equation (2) for calculating the shear strength contribution of the FRP. For a continuous FRP sheet, the shear strength contribution is given by

(9) $$V_{f}=w_{fe}t_{f}f_{fe}\;\sin^{2}\beta \leq \left(\frac{2\sqrt{f_{c}}b_{w}d}{3}-V_{s}\right)\;$$

where $w_{fe}\;$ can be computed as

(10) $$w_{fe}=d_{f}-2e^{\left\{6.134-0.58{\ln(t}_{f}E_{f})\right\}}$$

FIB [69] also provides an expression to estimate the shear strength contribution of the FRP, which is given as

(11) $$V_{f}=0.9\frac{2n_{f}t_{f}w_{f}E_{f}\epsilon_{fe}d_{f}}{s_{f}}$$

It is important to note that the ratio $w_{f}/s_{f}$ equals 1.0 in the case of continuous wraps. The effective strain for FIB in Equation (11) is denoted as

(12) $$\epsilon_{fe}=0.8{\left(0.17\frac{f_{cm}^{\frac{2}{3}}}{E_{f}\rho_{f}}\right)}^{0.30}\epsilon_{fu}\;$$

It is noted that $f_{cm}$ represents the mean compressive strength of concrete.

Sengun and Arslan [70] presented the following expression to determine the shear strength contribution by an FRP:

(13) $$V_{f}=\frac{2n_{f}t_{f}w_{f}E_{f}d_{f}}{s_{f}}\times 0.0018{\left(E_{f}\rho_{f}\right)}^{-0.84}\;$$

The comparison of predicted vs. experimental Lo-G contribution to shear strength of lightweight deep beams is presented in Table 4. It is evident that none of the existing models accurately predicted the shear strength contribution by Lo-Gs, which can be attributed to the significantly lower elastic modulus and tensile strength of Lo-G wraps compared to synthetic FRPs. The models by Khalifa et al., Triantafillou, and Sengun and Arslan tended to overestimate the experimental results, while the models by FIB and Zhang and Hsu underestimated them. The mean and standard deviations of the predictions are also presented in Table 4. Clearly, the predictions are marked by high standard deviations. In addition, the mean values of the prediction-to-experimental ratios are placed significantly away from 1.0. It is important to note that the presence of lightweight aggregates in combination with confinement that have a significantly lower elastic modulus and tensile strength than conventional FRPs are important factors that require separate models to determine shear strength contribution.

## 5. Conclusions

This study investigates the performance of lightweight aggregate deep beams strengthened with low-cost Lo-G wraps, establishing an alternate to the expensive synthetic FRPs. Since practical applications of FRPs to beams are restricted by the monolithic construction with slabs, the side-bonded application of Lo-G wraps is investigated in this work. Beams with Lo-G wraps fully wrapped around their perimeter are also explored. Moreover, two beams were also strengthened with a CFRP to provide a comparison with the efficiency of the proposed Lo-G wraps. The following conclusions are derived from the experimental findings.

Epoxy-based anchors provided significantly greater restrain against debonding than mechanical anchors. The findings suggest that while Lo-G wraps provide significant strengthening potential, proper anchorage placement and design are crucial for preventing debonding and ensuring the stability of the beam. Further research should focus on optimizing anchorage configurations and examining the behavior of beams with longitudinal openings to improve design models for practical applications.

The strengthened beams exhibited improved peak load capacity, with key findings indicating that beams without mechanical anchors performed better due to premature debonding in anchored specimens. Increasing the number of Lo-G wraps enhanced strength, while initial stiffness remained unchanged. Beams with epoxy anchors outperformed those with mechanical anchors or no anchors, though post-peak strength degradation was consistent across all beams. These results emphasize the importance of robust anchorage systems to prevent debonding and enhance performance.

The strengthened beams showed significant improvements in peak load and ultimate deflection. Side-bonded Lo-G wraps increased the capacity by 17.0% to 46.9%, while fully wrapped strip configurations improved it by 10.5% to 41.4% and CFRP strips by 15.4% to 42.7%. Lo-G wraps also enhanced ductility, with energy dissipation gains reaching 264.6% for side-bonded and 322.3% for fully wrapped configurations, exceeding peak capacity and deflection improvements. The side-bonded setup with two or three Lo-G wraps and epoxy layers outperformed fully wrapped 250 mm strips. However, mechanical anchors led to a poor performance due to premature debonding and the reduced cross-sectional area after pull-out. Additionally, reducing strip spacing to 50 mm negatively affected Lo-G wrap performance.

Beams strengthened with CFRP strips showed an 8.5% higher peak capacity and 32.8% greater dissipated energy compared to those strengthened with Lo-G wraps. Considering the cost difference, Lo-G wraps offer an effective alternative to CFRP strips. However, the long-term performance of Lo-G wraps remains to be investigated.

None of the existing models accurately predicted the shear strength contribution of Lo-G wraps, primarily due to their significantly lower elastic modulus and tensile strength compared to synthetic FRPs. The predictions exhibited high standard deviations, with mean prediction-to-experimental ratios significantly deviating from 1.0. The combination of lightweight aggregates and the lower mechanical properties of Lo-Gs highlights the need for distinct models to assess shear strength contribution effectively.

## Figures and Tables

**Table 1:** Summary of deep beams tested in this study.

Group	ID	Configuration	Anchorage	Quantity of Lo-G Wraps
1	DB-Ctrl	None	None	None
2	DB-2GSB-NA	Side-bonded	None	2
	DB-2GSB-EA	Side-bonded	Epoxy	2
	DB-3GSB-EA	Side-bonded	Epoxy	3
	DB-2GSB-MA	Side-bonded	Mechanical	2
	DB-3GSB-MA	Side-bonded	Mechanical	3
3	DB-1GW250-NA	Fully wrapped	None	1
	DB-2GW250-NA	Fully wrapped	None	2
	DB-3GW250-NA	Fully wrapped	None	3
	DB-1GW100-NA	Fully wrapped	None	1
	DB-2GW50-NA	Fully wrapped	None	2
	DB-3GW50-NA	Fully wrapped	None	3
4	DB-1CW50-NA	Fully wrapped	None	1
	DB-1CW250-NA	Fully wrapped	None	1

**Table 2:** Summary of the properties of strengthening materials.

Material	Thickness (mm)	Tensile Capacity (MPa)	Tensile Strain (%)	Elastic Modulus (GPa)
Lo-Gs	0.50	377	2.04	19
CFRP	0.13	5241	2.00	30

**Table 3:** Summary of dissipated energy, peak capacity, and ultimate deflection.

ID	Peak Capacity P (kN)	Increase in P (%)	Ultimate Deflection δ(mm)	Increase in δ(%)	Dissipated Energy E (kN-mm)	Increase in E (%)	Failure Mode
DB-Ctrl	290.1	-	2.9	-	475	-	Shear
DB-2GSB-NA	393.8	35.8	4.6	58.6	1287	170.9	Shear, debonding
DB-2GSB-EA	403.7	39.2	5.9	103.4	1597	236.2	Shear, debonding
DB-3GSB-EA	425.9	46.9	6.3	117.2	1732	264.6	Shear, debonding
DB-2GSB-MA	339.3	17.0	5.1	75.9	1150	142.1	Shear, debonding
DB-3GSB-MA	350.3	20.8	5.2	79.3	1178	148.0	Shear, debonding
DB-1GW250-NA	389.1	34.2	5.4	86.2	1377	189.9	Fracture, shear, debonding
DB-2GW250-NA	395.4	36.3	5.5	89.7	1417	198.3	Fracture, shear, debonding
DB-3GW250-NA	410.1	41.4	6.9	137.9	2006	322.3	Fracture, shear, debonding
DB-1GW100-NA	363.9	25.5	4.1	41.4	1144	140.8	Fracture, shear, debonding
DB-2GW50-NA	320.5	10.5	4.4	51.7	879	85.1	Fracture, shear, debonding
DB-3GW50-NA	349.0	20.3	4.3	48.3	1015	113.7	Fracture, shear, debonding
DB-1CW50-NA	334.6	15.4	5.6	93.1	1180	148.4	Fracture, shear, debonding
DB-1CW250-NA	413.8	42.7	7.1	144.8	1533	222.7	Fracture, shear, debonding

**Table 4:** Comparison of experimental vs. predicted shear strength contribution by Lo-G wraps.

ID	$V_{exp}$ (kN)	Predicted Strength									
		$V_{k}$ (kN)	$\frac{V_{k}}{V_{exp}}$	$V_{T}$ (kN)	$\frac{V_{T}}{V_{exp}}$	$V_{Z}$ (kN)	$\frac{V_{Z}}{V_{exp}}$	$V_{F}$ (kN)	$\frac{V_{F}}{V_{exp}}$	$V_{S}$ (kN)	$\frac{V_{S}}{V_{exp}}$
DB-2GSB-NA	51.9	101.6	2.0	101.3	2.0	23.5	0.5	10.7	0.2	53.8	1.0
DB-2GSB-EA	56.9	101.6	1.8	101.3	1.8	23.5	0.4	10.7	0.2	53.8	0.9
DB-3GSB-EA	68.0	111.4	1.6	134.6	2.0	26.0	0.4	14.3	0.2	57.4	0.8
DB-2GSB-MA	24.7	101.6	4.1	101.3	4.1	23.5	0.9	10.7	0.4	53.8	2.2
DB-3GSB-MA	30.2	111.4	3.7	134.6	4.5	26.0	0.9	14.3	0.5	57.4	1.9
DB-1GW250-NA	49.6	67.5	1.4	62.4	1.3	19.6	0.4	6.6	0.1	48.1	1.0
DB-2GW250-NA	52.7	101.6	1.9	101.3	1.9	23.5	0.4	10.7	0.2	53.8	1.0
DB-3GW250-NA	60.1	111.4	1.9	134.6	2.2	26.0	0.4	14.3	0.2	57.4	1.0
DB-1GW100-NA	37.0	49.2	1.3	47.0	1.3	26.6	0.7	7.5	0.2	67.7	1.8
DB-2GW50-NA	15.3	67.5	4.4	62.4	4.1	39.4	2.6	13.2	0.9	96.3	6.3
DB-3GW50-NA	29.5	88.2	3.0	82.8	2.8	43.7	1.5	17.6	0.6	102.7	3.5
Mean			2.2		2.3		0.4		0.3		2.0
Standard Deviation			1.2		1.2		0.3		0.2		1.5

Note: $V_{k}$, $V_{Z}$, $V_{T}$, $V_{F}$, and $V_{S}$ represent the shear contribution predicted by the models by Khalifa et al., [66] Zhang and Hsu [68], Triantifillou [67], FIB [69], and Sengun and Arslan [70].

## Data Availability

The original contributions presented in this study are included in the article. Further inquiries can be directed to the corresponding author.
